# Effect of Mindfulness Breathing Meditation on Depression, Anxiety, and Stress: A Randomized Controlled Trial among University Students

**DOI:** 10.3390/healthcare11010026

**Published:** 2022-12-22

**Authors:** Maria Komariah, Kusman Ibrahim, Tuti Pahria, Laili Rahayuwati, Irman Somantri

**Affiliations:** 1 Department of Fundamental Nursing, Faculty of Nursing, Universitas Padjadjaran, Sumedang 45363, Indonesia; 2 Department of Medical-Surgical Nursing, Faculty of Nursing, Universitas Padjadjaran, Sumedang 45363, Indonesia; 3 Department of Community Health Nursing, Faculty of Nursing, Universitas Padjadjaran, Sumedang 45363, Indonesia

**Keywords:** anxiety, depression, mindfulness, stress, students

## Abstract

Background: The COVID-19 outbreak has caused various changes in all aspects of human life, including the educational system. These changes have forced students to undertake an adaptive process that has inevitably affected aspects of their life and psychological well-being. Adaptation of learning into online forms in universities, including nursing, triggers depression, stress, and anxiety. The high number of incidences of stress, anxiety, and depression in undergraduate students throughout the pandemic has made it important to prevent and deal with health approaches, such as mindfulness therapy. Objective: This research intended to examine whether an intervention based on mindfulness was effective and had the potential to become an interference to reduce anxiety, stress, and depression in Indonesian university students. Methods: This research applied a randomized controlled trial. One hundred and twenty-two students from Universitas Padjadjaran and other provinces in Indonesia participated in this study, with sixty-one students in each group. A pretest and a post-test were administered before and after the intervention using the Depression Anxiety Stress Scales (DASS-42). The intervention was carried out for 4 weeks with 15 min practice in each session. Results: In this study, there was an effect of mindfulness breathing meditation on decreasing the mean scores for depression, anxiety, and stress in the intervention group. However, only stress (*p* = 0.007) and anxiety (*p* = 0.042) showed a significant difference in the post-test results of the intervention and control groups. In addition, there was no difference in the scores of stress, anxiety, and depression for the pre–post-tests in each group based on religion. Conclusion: Mindfulness breathing meditation has an impact on reducing stress and anxiety in students, so it could be applied to all university students in order to develop psychosocial status and mindful attentiveness to one’s needs.

## 1. Introduction

The arrival of the COVID-19 pandemic has impacted the physical well-being and mental health of individuals around the world. Indonesia appealed to its people to carry out social distancing and physical distancing according to the WHO protocol by imposing large-scale social restrictions, including in the education sector, by enforcing all educational activities to be carried out online as an effort to reduce mass crowds and prevent the transmission of COVID-19. The transition to online learning poses special difficulties because the learning methodology requires adaptation [1], so the learning process that is experienced will be different from the face-to-face learning that is usually conducted. This may impact the psychological condition of students because many students struggle with mental health issues during their college years. The restrictions imposed by the COVID-19 pandemic and the shift to online learning environments may become even more apparent for students’ emerging mental health issues [2]. Adapting learning to online university forms triggers depression, anxiety, and stress.

A survey conducted in China at the start of the outbreak showed that 53.8% of respondents experienced modest to extreme psychological effects, 16.5% had modest to extreme depressive symptoms, 28.8% experienced modest to extreme anxiety symptoms, and 8.1% experienced modest to extreme levels of stress [3]. Another study in Brazil showed that the majority of college students surveyed exhibited symptoms of mild to severe depression (60.5%), anxiety (52.5%), and stress (57.5%) [4]. A similar previous study in Lebanon concluded that the sudden change in learning methods during COVID-19 caused a stressful workload, which began to cause anxiety and depressive symptoms among undergraduate students [5].

Research in Korea reported that depression is the most critical cause of social problems or suicide. To determine efficacious depression anticipation schemes in the US, the American College Health Association (ACHA) actualized a national student mental health need and promoted it [6,7]. Depression also occurs in students in Indonesia. Research by Santoso et al. [8] on 148 nursing students in Central Java, Indonesia, found that students experienced mild mood disorders (25.7%), low depression (8.1%), moderate depression (0.7%), severe depression (12.2%), and extreme depression (0.7%).

Depression impacts around 67% of college students with anxiety, which becomes the main predictor of depression for nursing students [6,9]. Garcia-Gonzalez et al. [10] concluded that nursing students had high levels of anxiety during the first and fourth weeks of COVID-19 confinement. Besides that, research conducted on Chinese students throughout the COVID-19 pandemic discovered that around 25% of respondents experienced anxiety symptoms positively in proportion to increasing considerations about academic postponement, the economic impact of the pandemic, and the impact on everyday life [11]. Furthermore, a study by Young Minds showed that 83% of global young participants were positively certain that the pandemic exacerbated preceding mental health states, primarily because of the prolonged school time, loss of daily activities, and limited social contact [12]. Anxiety is influenced by emotion-oriented coping mechanisms, such as emotional responses. Therefore, it is suspected that an adaptive coping strategy, for instance, mindfulness-based intervention (MBI), can lessen depression, stress, and anxiety [13,14].

Apart from depression and anxiety, stress is a psychological problem that is prone to occur in students during a pandemic. Stress is an essential psychosocial aspect of learning activities that can affect students’ academic accomplishment and well-being [15]. The high prevalence of stress among students throughout the COVID-19 pandemic was reported by Aristovnik et al. [16], who stated that the COVID-19 pandemic resulted in the closure of 1.59 billion educational institutions from 194 countries in the world, resulting in a dynamic alteration in the educational world of students, staff teachers, and families involved. Another study by Son et al. [17] found a rise in stress and anxiety levels throughout the COVID-19 pandemic social restrictions. A total of 173 out of a total of 195 students said that the stress experienced during the pandemic affected their concentration during study. Most of them, namely as much as 48%, thought that the situation at home was the biggest distraction that made it difficult for students to concentrate during online learning. In addition, another influencing factor was that the long period spent in front of a computer screen during online learning had side effects concerning vision and headaches and limited students’ social interactions. Another study also stated that during the pandemic, society, including higher education students, experienced an increase in emotional disorders, such as stress, insomnia, frustration, and unstable irritability, causing psychological disorders, for instance, depression, anxiety, behavioral changes, and post-traumatic stress disorder (PTSD) [18].

The condition of psychological disorders experienced by students requires intervention that can minimize these disorders. Health experts have found various interventions to prevent and manage stress, depression, and anxiety events, one of which is therapy mindfulness [6,19]. Mindfulness-based intervention is currently becoming a popular therapy among the world community. Mindfulness is said to be a complete therapy that can minimize stress levels and improve psychological well-being because it includes various components of therapy, such as yoga, deep breathing techniques, focusing attention, and acceptance without judgment. Mindfulness is considered one of the core components of the meditation process, which includes two key elements, namely sustaining focus on the immediate experience and demonstrating acceptance of the experience [20]. Mindfulness therapy is effective and flexible because it can be applied to all ages, from children to the elderly, without any contraindications. In addition, mindfulness therapy is also very easy to do wherever and whenever according to the time desired by the individual [21].

In addition, in a study conducted by Spears [22], mindfulness is said to be a therapy that aims to decrease the level of stress, depression, and anxiety experienced by individuals universally. This means that mindfulness therapy can be given to individuals from various religions, beliefs, and ethnic and cultural backgrounds. Mindfulness is believed not to conflict with certain teachings or beliefs so that individuals can undergo this therapy without worry [22]. Barnes et al. [23] said that mindfulness is a therapy that involves the mind and body in an integrated manner to help individuals find calm when dealing with stressors, challenges, or illnesses. In the same study, it was also explained that mindfulness is not a health intervention but a skill that each individual should have [23]. Meanwhile, in Black’s research, it was stated that mindfulness is a systematic procedure that sharpens and awakens the natural human capacity to be fully present in the present, welcomes and acknowledges, as well as possible, all the series of events that have occurred, whether pleasant, sad, or neutral events, and produce results regarding wisdom in behavior [24]. Therefore, it can be concluded that mindfulness is a therapy that involves the mind as well as the body to be fully present and accept the present experience to gain calm and wisdom in dealing with stressors.

Meanwhile, another study conducted on 131 students from several universities in America stated that mindfulness therapy did not significantly impact students’ stress levels [25]. In addition, based on research on 288 students, mindfulness did not show a positive reaction to both reducing stress and student welfare [26]. This shows that there are quite contradictory differences in results.

A large number of events of stress, depression, and anxiety in global students, especially during the COVID-19 pandemic, make it important to prevent and deal with health approaches, such as mindfulness therapy. It is critical to consider the effects of these interventions in order for students to make evidence-based decisions concerning mental healthcare at university. While mindfulness is widely available, it is unknown whether it has a beneficial effect on the mental health of university students. Therefore, this study intended to examine whether an intervention based on mindfulness was effective and potentially an intervention to decrease stress, depression, and anxiety in university students.

## 2. Materials and Methods

### 2.1. Study Design

This research was a randomized controlled trial. Students from universities in various provinces in Indonesia, covering all provinces in Java, Sumatra, and Borneo island, took part in this study. Moreover, this study was registered and approved by TCTR with register number TCTR2022080800.

### 2.2. Recruiting and Sampling

A total of 122 students from 16 universities in Indonesia participated in this study. Recruitment of participants was carried out through open recruitment with information dissemination assisted by posting the flyer on social media. Each potential participant was reached through a cell phone communication application. A number of these students were selected based on the established criteria. The inclusion criteria in this study were: (1) Indonesian university students from all provinces in Java, Sumatra, and Borneo island who were willing to join the program and (2) adults (aged ≥18 years old) as the inclusion criteria. There was also an exclusion criterion including students with severe mental disorders. Before conducting the research, participants were given research information or informed consent, which was provided online through the Google Form. The informed consent was limited to one person, contained the respondent’s willingness to participate, and participants could freely choose to participate or not in this research. This recruitment process was carried out in January 2022.

Participants in this study were divided into two groups, namely the intervention and control groups. The size of the sample uses the G* power formula by determining the significance level (α = 0.05), effect size (d = 0.80), and power (80%). From the calculation based on the formula, the sample size was 26 people in each group. As for this study, there were 122 respondents, and none dropped out, so each group consisted of 61 students. Randomization with a simple random sampling approach was carried out to determine the distribution of groups for participants. This procedure was carried out by contactless enumerators of participants who were blindly selected by randomization minimization software 12.0. The results of this randomization then determined the placement of participants and whether they entered the intervention or control group.

### 2.3. Data Collection

Before the intervention was started, all participants’ stress, anxiety, and depression were measured with the DASS-42, which was regulated through Google Forms. The DASS-42 includes 42 articles assessing the level of stress, anxiety, and depression [27]. The outcome of the study including the level of depression, anxiety, and stress was assessed using the DASS instrument. DASS is a psychological measurement instrument that measures depression, stress, and anxiety scale in individuals. Developed by Lovibond and Lovibond (1995), it consists of 42 question items in which each measurement scale consists of 14 items [28]. The instrument used in this study was DASS-42, which has been translated into an Indonesian version [29]. A validity test by Damanik and Rusli showed that the 42 items were valid. The reliability values obtained for the Indonesian language DASS instrument for the depression, anxiety, and stress scales were 0.872, 0.806, and 0.816, respectively [30]. The result indicated that the instrument is valid and reliable to use.

Research activities ranging from pretest measurements, carrying out the intervention, and the post-test were carried out from February to March 2022. The participants were then requested to join a WhatsApp group (WAG) initiated by researchers, which described the comprehensive protocol of mindfulness breathing meditation and provided support with video practice guidelines and tutorials. The intervention was carried out online through a Zoom meeting. Therefore, there were research assistants who assisted in the implementation of interventions during the research with blind group placement. The participants were required to exercise mindfulness breathing meditation every day for 4 weeks with 15 min duration on each day. In the first 2 weeks, the intervention was carried out with guidance through Zoom meetings. Meanwhile, in the following 2 weeks, the participants conducted their intervention without guidance from the research assistant. They were permitted to choose the most convenient times and places to exercise. To make sure that their exercise followed the guidelines, they were required to complete a reflective form provided by the researchers. Every week, participants were given reminders regarding the implementation of the intervention. The respondents practiced breathing meditation for 4 weeks, during which the researchers actively motivated and maintained contact with the participants through WAG chats. Unlike the intervention group, the control group did not carry out activities related to mindfulness breathing meditation during those 4 weeks. The researcher conveyed that the participants should continue to carry out activities as usual, both at home and elsewhere. Even so, the control group still received video practice guidelines and tutorials for mindfulness breathing meditation after a series of studies.

After 4 weeks, a follow-up was carried out, and the participants were required to fulfill the self-administered DASS-42 questionnaire via Google Forms as a post-test. The DASS-42 was directed by the researchers as a pretest survey before and as a post-test survey after the 4 weeks. During the administration of the intervention, no participants dropped out, so the number of students in each group was constant (*n* = 61) from the pretest to the post-test.

**Figure 1:**
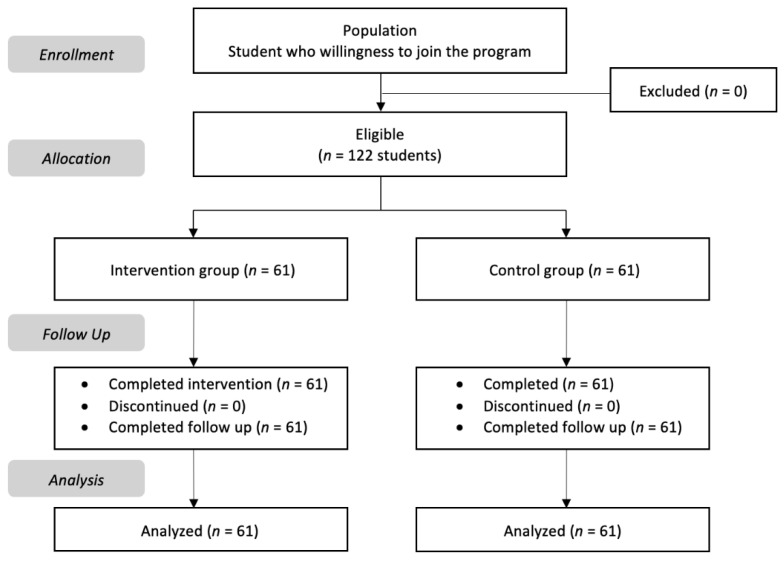
Consort study flow diagram.

The data for analysis used in the first measuring is given in Figure 1, which shows the consort study flow chart.

### 2.4. Statistical Analysis

Data were collected from Google Forms and relocated to SPSS software version 24 (IBM Corp., Armonk, NY, USA). SPSS was used to analyze the demographic data and also baseline and intervention data for stress, anxiety, and depression. In this study, the analysis used univariate and bivariate analysis. A univariate analysis technique was conducted to determine the distribution, frequency, and percentage of respondents’ characteristics, which included age, gender, and religion, besides anxiety, stress, and depression levels with the criteria: 0–14 = normal, 15–18 = mild stress level, 19–25 = moderate stress level, 26–33 = severe stress level, and >34 = very severe stress level; 0–9 = normal, 10–13 = mild depression level, 14–20 = moderate depression level, 21–27 = severe depression level, and >28 = very severe depression level; 0–7 = normal, 8–9 = mild anxiety level, 10–14 = moderate anxiety level, 15–19 = severe anxiety level, and >20 = very severe anxiety level, respectively.

The second analysis was a bivariate analysis. In this study, an independent *t*-test was performed to analyze the comparison of the intervention and control groups. We also used a one-way analysis of variance (ANOVA) to determine the effect of other variables on the effectiveness of mindfulness interventions on stress, anxiety, and depression.

### 2.5. Ethical Clearance

Ethical clearance for this research was granted by the ethics research committee of Universitas Padjadjaran with letter no. 1062/UN6.KEP/EC/2021. Participants involved in this study were instructed about the research purposes, and informed consent was acquired as a legal requisite.

## 3. Result 

### 3.1. Characteristics of Participants

A total of 122 participants fulfilling the criteria were included in this study (mean age, 20.30 ± 1.116 in the intervention group and 22.42 ± 3.672 in the control group; most respondents were women, 51 (83.60%) in the control group and 42 (68.9%) in the intervention group; and majority religion was Islam with 54 respondents). Table 1 displays the university students’ sociodemographic variables; there were no noteworthy differences between the control and intervention groups, except for the age variable.

### 3.2. Baseline and Post-Intervention Stress, Anxiety, and Depression of Participants

Based on the analysis of stress, anxiety, and depression in the pre- and post-tests, in Table 2, it can be seen that there is a difference in mean and standard deviation in each group. From these data, it was also found that there was no significant differences in each group for the baseline data for stress (*p* = 0.862), anxiety (*p* = 0.664), and depression (*p* = 0.363). As for the post-test, the test results showed that there were significant differences between the post-test categories of the two groups in terms of stress (*p* = 0.007) and anxiety (*p* = 0.042). Whereas in depression, the results showed that there was no significant difference in the post-test data of the two groups. Even so, in the intervention group, there was a decrease in mean and SD for all variables. Thus, even though there was no significant change in the depression variable, the interventions were successful in reducing the average depression score in that group.

### 3.3. Frequency Distribution of Participants’ Levels of Stress, Anxiety, and Depression

Based on an analysis of the frequency distribution of stress, anxiety, and depression, it was found that respondents who participated in this study were divided into five categories, namely normal, mild, moderate, severe, and extremely severe, in each of the two groups’ variables. Before giving the intervention, apart from being at a normal level, the other categories that were mostly found for stress occurred in the moderate category (60.0%) in the intervention group and severe (55.6%) in the control group, anxiety occurred at a moderate level for the intervention group (55.9%) and control (44.1%), while depression occurred at a moderate level for the intervention group (46.2%) and control (53.8%). After giving the intervention, stress, anxiety, and depression categories were dominated by normal levels in the intervention group for stress (44.3%), anxiety (42.3%), and depression (57.7%), and in the control group for stress (55.7%), anxiety (57.7%) and depression (58%). These results are shown in Table 3.

### 3.4. Age and Religion Factors on Participant’s Interventions

Table 4 shows the results of testing the age and religion categories regarding their influence on successful interventions. These results are presented in the form of data on the average and standard deviation of each variable for each group, followed by the *p*-values for the post-test of the two groups with one-way ANOVA processing. Based on the test results, it was found that there was no difference in the scores for stress, anxiety, and depression for the pre- or -post-tests, either in the treatment group or the control group based on age (*p* = 0.63, 0.457, and 0.573) and religion (*p* = 0.568, 0.505, 0.534).

## 4. Discussion

### 4.1. Principal Finding

At the baseline data of the intervention and control groups, there was no significant difference between the two groups for stress, anxiety, and depression. Meanwhile, in the post-test data, it was found that there were significant differences between groups for stress and anxiety.

From the findings, the data showed that, compared with the control group, the intervention group that received the mindfulness therapy program resulted in a reduction in depression, anxiety, and stress. In this study, which is about the effectiveness of mindfulness therapy on depression, anxiety, and stress, it appears that the average score of the intervention group decreased more than the average score of the control group, in which the control group experienced a slight change in score. This finding is similar to a study by Ibrahim et al. [31], in which MBSR effectively increased psychological well-being. Additionally, a study by Nyklicek et al. [32] described that an MBSR program consisting of 90–120 min of instruction for only three sessions per week effectively reduced depression, anxiety, and stress in 107 percutaneous coronary patients.

Although it resulted in a higher mean score reduction in stress, anxiety, and depression in the intervention group, the post-test results for depression in the control and intervention groups showed no significant differences. Only stress and anxiety showed a significant difference in the post-test results between the intervention and control groups. Thus, the most significant change is indicated by stress, which is shown by a *p*-value that is smaller than the *p*-value of anxiety. The results are similar to Chiodelli et al.’s study that stress was the variable that had a significant difference. The use of nonclinical populations in the research has the potential to affect the effectiveness of mindfulness intervention. This could be because the reduction is significantly more likely to be experienced in populations who already experience high levels of depression and anxiety [33]. The same finding was also reported by Chen et al. [34]—decreasing levels in anxiety scores were significantly greater in the MBSR group than in the control group. However, there was not a significant result for depression levels. In connection with these matters, the respondents included in this study consisted of students of various ages and religions. However, the test results showed that these two aspects did not significantly influence the intervention for the two groups. Meanwhile, if viewed from a psychological condition, the respondents who were included in this study were not only respondents with high levels of stress, depression, and anxiety, but all levels were included, so this might also affect the effectiveness of the interventions carried out.

According to Chen et al. [34], the reason for this negative finding is that the MBSR program was only conducted as a short-term intervention, which was only for 7 consecutive days. In this study, the time of the intervention was carried out for 4 weeks. In a previous study on MBSR with 32 nursing students, an MBSR program was conducted for 8 weeks [35]. Moreover, in another study in which an MBSR was carried out for 2.5 h every week for 8 consecutive weeks, it showed a significant reduction in depression and stress in 83 patients with chronic disease [36]. Compared with other studies that show significant results on depression, stress, and anxiety, the intervention time in this research tends to be less. This is because some articles indicate an intervention duration of 8 weeks, while in this study, the intervention only lasted 4 weeks. Thus, the researchers suspect that the length of time an intervention is given can be one of the factors that increasingly influence the effectiveness of the mindfulness intervention.

Stress is a pressure that is felt by an individual as a result of an imbalance between the demands of their needs and the capacity they have, so it has an impact on the individual’s biopsychological condition. Students belong to a group that is prone to experiencing stress related to academic and nonacademic responsibilities, such as relationships with family and students’ social environment. Not only stress but anxiety and depression were also felt by students. Those psychological problems, if otherwise not handled properly, can result in various negative impacts that affect the physical and psychological well-being of students. Therefore, efforts to reduce stress, anxiety, and depression experienced by students need to be carried out so that it also has an impact on the optimization of the learning process during lectures. The results of this study indicate that mindfulness-based therapy can be an effective intervention to reduce student stress and anxiety. Mindfulness is a therapy that involves integrating the mind and body to fully focus, present oneself, and accept left-hand experiences to gain calm, wisdom, and peace in dealing with stressors.

In this study, the mindfulness therapy used is as a type of breathing meditation, which is a type of MBSR that is performed by focusing on abdominal breathing, along with feeling the sensations felt in the body, mind, and feelings without paying attention to things other than the sensations or emotions felt [37]. This exercise is performed in an upright sitting position but remaining relaxed, with the chest open for some time in a state of silence. According to Lutz (in Kropp and Sedlmeier) [38], Nagas meditation includes three basic abilities, namely monitoring the stimulus or sensation felt without distracting focus, the ability to separate oneself from possible distractions, and the ability to focus fully on the object or sensation that is felt.

From several findings, it was found that significant results in reducing stress levels were obtained from participants who participated in face-to-face and online MBSR therapy using the Mindful Skills for Student (MSS) application. Therefore, the combination of offline and online MBSR intervention was effective in helping participants manage the stress they experienced. Based on previous research, it was stated that internet-based mindfulness interventions have the potential to meet the needs of participants because they are more accessible and flexible so that they can be integrated with healthcare facilities to help manage stress, depression, and anxiety [39,40,41,42].

### 4.2. Limitations

The research findings obtained by several participants in the study conveyed the factors that became obstacles while carrying out mindfulness breathing meditation. The biggest obstacle that occurred during the intervention was the low level of public awareness and sociocultural influences. For instance, in this study, awareness was still very low among the campus community, and where participants lived resulted in mindfulness interventions not being widely known. This was indicated by a lack of public knowledge about the existence of mindfulness therapy and a lack of facilities and infrastructure in the campus environment and where participants live that provide information related to mindfulness. Other inhibiting factors are from a social and cultural perspective. Participants felt that the practice of mindfulness was not familiar in their social and cultural environment because, since childhood, there was no one that required them to behave mindfully. In this study, the age of the respondents was not homogeneous, but the practice of mindfulness can be carried out by various ages.

In addition, the respondents who were included in this study ranged from respondents with normal levels of stress, anxiety, and depression, so the possible symptoms they experienced were not too severe for some respondents. Besides that, the control group was only contacted individually for each respondent, and the researcher did not administer any treatment regarding mindfulness interventions that could be obtained with media or other methods that were different from the intervention group when the intervention group received the MBSR intervention.

### 4.3. Implications for Clinical Practice

The current result of our study indicates that mindfulness breathing meditation can be a promising strategy to efficiently reduce the levels of stress, depression, and anxiety. The fact that this meditation can be carried out at any time or any place also makes it a very cost-effective wellness intervention that can be accessed by everyone universally. Furthermore, this study provides a rationale for including this therapy in any nursing courses as one of the effective ways to make students relax. This intervention can be carried out using MP3 or MP4 video media files to guide the implementation of practical activities so that it can also be carried out by students independently. With the integration of an application for mindfulness implementation on campus, it is hoped that it will be able to increase students’ abilities and concentration so that each intervention that is carried out can be more effective.

## 5. Conclusions

This study showed that mindfulness breathing meditation provides a reduction in scores of stress, depression, and anxiety among university students in Indonesia. However, an effective reduction occurred for stress and anxiety at the 4-week follow-up. Mindfulness breathing meditation is a nonpharmacological method, which means it can be implemented virtually at any time and any place. The use of mindfulness breathing meditation could be applied to all university students and so forth to develop psychosocial status and mindful attentiveness to one’s needs.

From this study, we recommend a future study to conduct research about the factors that influence the effectiveness of giving mindfulness breathing meditation therapy. We also recommend that future researchers provide treatment to the control group related to the intervention during the research, for example, providing intervention information with poster media. This is intended so that all participants in the study receive the same treatment regarding the intervention. Additionally, a future study to assess the long effect of 6–12 months of follow-up is needed in the new normal era.

## Figures and Tables

**Table 1:** Demographic of participants.

Characteristic	Control Group	Intervention Group	*p*-Value
Gender			
Female	51 (83.60%)	42 (68.90%)	0.089
Male	10 (16.40%)	19 (31.15%)	
Religion			
Islam	54 (49.1%)	56 (50.9%)	0.361
Christian	5 (50%)	5 (50%)	
Buddhism	2 (100%)	0 (0%)	
Age	20.30 ± 1.116	22.42 ± 3.672	0.000

**Table 2:** Baseline and post-intervention stress, anxiety, and depression of participants.

Variable		Intervention Group M (SD)	Control Group M (SD)	*p*-Value
Stress	Baseline	16.31 (9.807)	16.00 (10.00)	0.862
	Post	10.07 (6.957)	14.03 (8.77)	0.007
Anxiety	Baseline	14.08 (8.737)	13.39 (8.74)	0.664
	Post	8.46 (5.448)	10.66 (6.32)	0.042
Depression	Baseline	13.93 (9.631)	12.36 (9.42)	0.363
	Post	7.54 (7.309)	9.46 (7.70)	0.161

**Table 3:** Frequency distribution of participants’ levels of stress, anxiety, and depression.

Variable	Intervention Group		Control Group	
	Baseline	Post	Baseline	Post
**Stress**				
**Normal**	32 (51.6%)	44 (55.7%)	30 (48.4%)	35 (44.3%)
**Mild**	8 (50.0%)	8 (42.1%)	8 (50.0%)	11 (57.9%)
**Moderate**	8 (40.0%)	7 (46.7%)	12 (60.0%)	8 (53.3%)
**Severe**	10 (55.6%)	0 (0.0%)	8 (44.4%)	6 (100.0%)
**Extremely** **severe**	3 (50.0%)	2 (66.7%)	3 (50.0%)	1 (33.3%)
**Anxiety**				
**Normal**	15 (50.0%)	30 (57.7%)	15 (50.0%)	22 (42.3%)
**Mild**	6 (50.0%)	7 (46.7%)	6 (50.0%)	8 (53.3%)
**Moderate**	15 (44.1%)	17 (54.8%)	19 (55.9%)	14 (45.2%)
**Severe**	9 (60.0%)	4 (28.6%)	6 (40.0%)	10 (71.4%)
**Extremely** **severe**	16 (51.6%)	3 (30.0%)	15 (48.4%)	7 (70.0%)
**Depression**				
**Normal**	21 (43.8%)	29 (58.0%)	27 (56.2%)	21 (42.0%)
**Mild**	13 (52.0%)	18 (62.1%)	12 (48.0%)	11 (37.9%)
**Moderate**	14 (53.8%)	10 (37.0%)	12 (46.2%)	17 (63.0%)
**Severe**	6 (60.0%)	2 (22.2%)	4 (40.0%)	7 (77.8%)
**Extremely** **severe**	7 (53.8%)	2 (28.6%)	6 (46.2%)	5 (71.4%)

Note: data are expressed as frequency (*n*) and percentage (%) for each level of variables.

**Table 4:** Mean scores (standard deviations) based on age and religion of participants.

Variable	Baseline		Post		*p*-Value *
	IG (M, SD)	CG (M, SD)	IG (M, SD)	CG (M, SD)	
Age (years)					
Stress					
<21	14.66 (9.152)	21.83 (7.627)	10.00 (6.958)	14.83 (9.827)	0.63
21	18.82 (11.775)	15.19 (6.322)	11.12 (7.960)	13.05 (8.851)	
>21	18.00 (7.714)	15.47 (11.909)	8.33 (4.975)	14.50 (8.760)	
Anxiety					
<21	12.60 (8.388)	17.33 (5.007)	8.23 (5.180)	13.50 (6.504)	0.457
21	15.59 (10.381)	13.10 (6.503)	9.71 (6.659)	11.05 (7.619)	
>21	17.00 (5.831)	12.88 (10.304)	7.00 (3.742)	9.91 (5.384)	
Depression					
<21	12.66 (9.434)	16.67 (8.335)	7.74 (6.423)	11.83 (6.969)	0.573
21	16.76 (11.306)	11.67 (6.102)	8.35 (10.000)	11.10 (9.633)	
>21	13.56 (6.126)	12.03 (11.156)	5.22 (4.206)	8.03 (6.264)	
Religion					
Stress					
Islam	17.20 (9.727)	16.11 (10.101)	10.69 (7.012)	14.41 (8.891)	0.568
Christian	10.00 (8.337)	14.80 (9.731)	4.80 (2.683)	9.80 (6.496)	
Buddhism	8.00 (9.899)	-	6.50 (9.192)	-	
Anxiety					
Islam	14.89 (8.801)	13.43 (8.932)	9.00 (5.425)	10.71 (6.387)	0.505
Christian	8.20 (4.868)	13.00 (6.892)	3.80 (2.168)	10.00 (6.205)	
Buddhism	7.00 (8.485)	-	5.50 (7.778)	-	
Depression					
Islam	14.50 (9.743)	12.63 (9.680)	7.93 (7.581)	9.55 (7.865)	0.534
Christian	9.40 (7.436)	9.40 (5.550)	3.40 (1.140)	8.40 (6.025)	
Buddhism	10.00 (12.728)	-	7.50 (7.778)	-	

Note: * One-way ANOVA test; IG, = intervention group; CG = control group; M = mean; SD = standard deviation.

## Data Availability

Not applicable.
